# Supplementary material for: Phase transformation in lead titanate based relaxor ferroelectrics with ultra-high strain
Source: Nat Commun. 2025 Feb 18;16:1720. doi: 10.1038/s41467-025-56920-9 (PMC11836266; doi:10.1038/s41467-025-56920-9)
Supplement: Supplementary file 1 — Supplementary Information [file 41467_2025_56920_MOESM1_ESM.pdf]

Supplementary Materials for

Phase transformation in lead titanate based relaxor ferroelectrics with ultra-high strain

Hangfeng Zhang[1], Zilong Li[1], Yichen Wang[1], A. Dominic Fortes[2], Theo Graves Saunders[3], Yang Hao[3*], Isaac Abrahams[4*], Haixue Yan[1*], Lei Su[1*]

[*]Corresponding authors. Email: y.hao@qmul.ac.uk, i.abrahams@qmul.ac.uk, h.x.yan@qmul.ac.uk, l.su@qmul.ac.uk.

The PDF file includes:

Supplementary method
Figs. S1 to S24
Tables S1 to S4
References

Supplementary method

Evaluation of spontaneous polarisation

In the T and O phases, the spontaneous polarisation (P_s) is linked to a total dipole moment oriented parallel to the *c*-axis. This dipole moment can be quantified by evaluating the displacement of individual atoms within the unit cell away from their position in an ideal centrosymmetric arrangement as shown in (Eqn. 1) [1,2] :

$$P_s = \sum_i (m_i \times \Delta \boldsymbol{x_i} \times Q_i e)/V \qquad (1)$$

where m_i represents the number of atoms of type *i* in the unit cell, Δx_i is the atomic displacement away from the ideal centrosymmetric position, $Q_i e$ is the ionic charge and *V* is the unit cell volume. It is important to note that the position of Pb/Er was fixed at the unit cell origin (0, 0, 0) during the structure refinement. Therefore, the calculated displacements and their contribution to the total polarisation are all relative to this reference point.

Supplementary Fig. 1 Fitted XRD profiles of studied compositions in the $\mathbf{Er_{0.025}Pb_{0.9625}(Mg_{0.33}Nb_{0.67})_{1-\mathit{x}}Ti_{\mathit{x}}O_3}$ system: (**a**) $x = 0.26$, (**b**) $x = 0.28$, (**c**) $x = 0.30$, (**d**) $x = 0.32$ and (**e**) $x = 0.34$. Reflection positions are indicated by markers with those for both *P*4*mm* (lower) and *Amm*2 (upper) shown in the multiphase refinements (**d**, **e**).

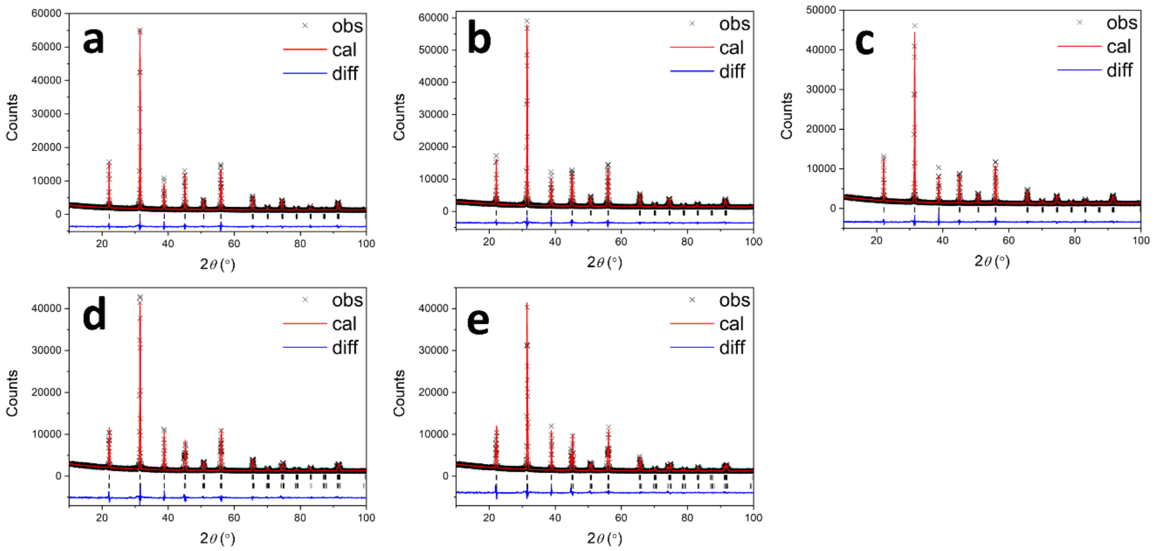

Supplementary Fig. 2 SEM images of ceramic fracture surfaces for compositions in the system $Er_{0.025}Pb_{0.9625}(Mg_{0.33}Nb_{0.67})_{1-x}Ti_xO_3$: (**a**) $x = 0.26$, (**b**) $x = 0.28$, (**c**) $x = 0.30$, (**d**) $x = 0.32$ and (**e**) $x = 0.34$.

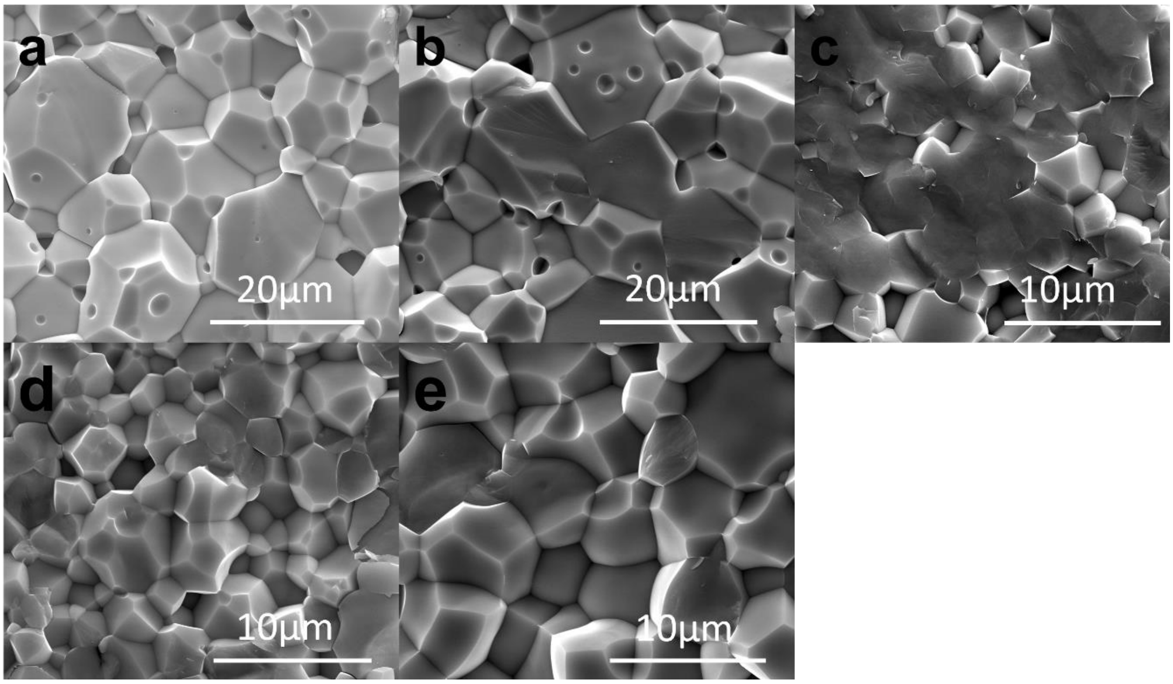

Supplementary Fig. 3 Temperature dependence of dielectric properties for studied compositions in the system $Er_{0.025}Pb_{0.9625}(Mg_{0.33}Nb_{0.67})_{1-x}Ti_xO_3$: (**a**) $x = 0.26$, (**b**) $x = 0.28$, (**c**) $x = 0.30$, (**d**) $x = 0.32$ and (**e**) $x = 0.34$. All compositions show one dielectric permittivity peak, accompanied by a corresponding dielectric loss peak, indicating the transition from ferroelectric to paraelectric states at the Curie temperature, T_c.

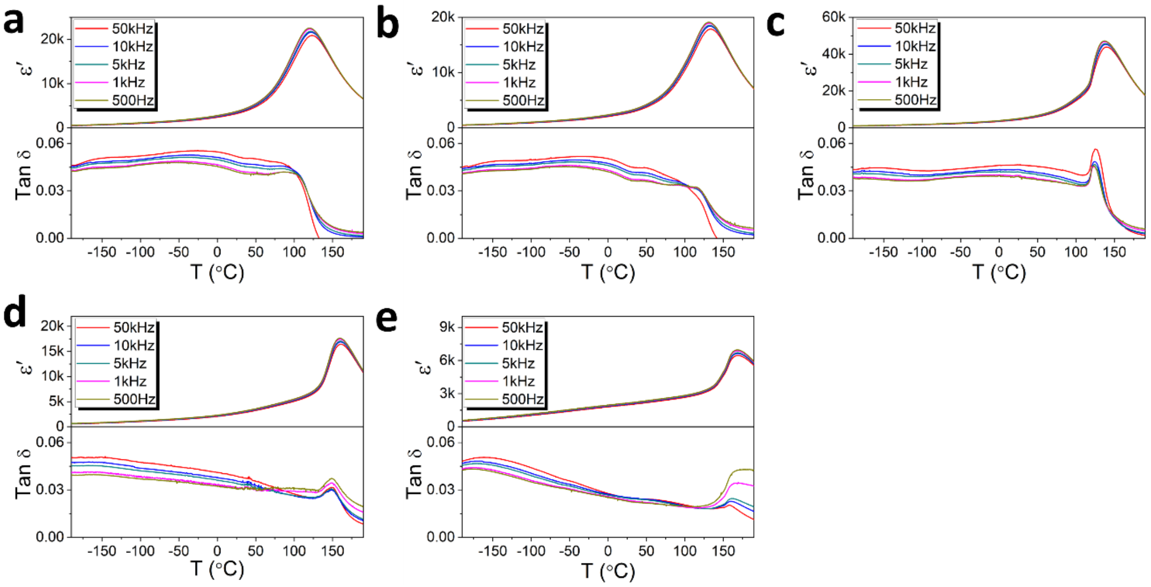

Supplementary Fig. 4 *I-E* (left), *P-E* (middle) and *S-E* (right) loops for compositions in the system $Er_{0.025}Pb_{0.9625}(Mg_{0.33}Nb_{0.67})_{1-x}Ti_xO_3$ measured at room temperature. In all compositions, typical ferroelectric *P-E* hysteresis loops are observed with two current peaks corresponding to the coercive field in the *I-E* loops, and a butterfly shape in the *S-E* loops. Unlike other relaxor ferroelectrics, all the *S-E* butterfly loops are almost perfectly symmetrical, which indicates minimal defect concentrations in the material (either cation or oxygen vacancies) occurred during the high temperature sintering process [3,4]. The coercive field value gradually increases with increasing titanium concentration, from 0.45 kV mm^{-1} for the $x = 0.26$ composition to 0.8 kV mm^{-1} for the $x = 0.34$ composition, with the saturation polarisation value of all the compositions close to 0.4 C m^{-2}. Due to their low coercive electric field values, all the samples show saturated polarisation and very high strain at a relatively low electric field of 1 kV mm^{-1}. The strain at 5 kV mm^{-1} increased with increasing x-value from *ca*. 0.66% for the $x = 0.26$ composition to *ca*. 3.34% for the $x = 0.30$ composition, then decreased to 2.5% and 0.97% for the $x = 0.32$ and 0.34 compositions, respectively. The bipolar strain is well above 2% for $x = 0.30$ and 0.32 compositions under the measured electric fields.

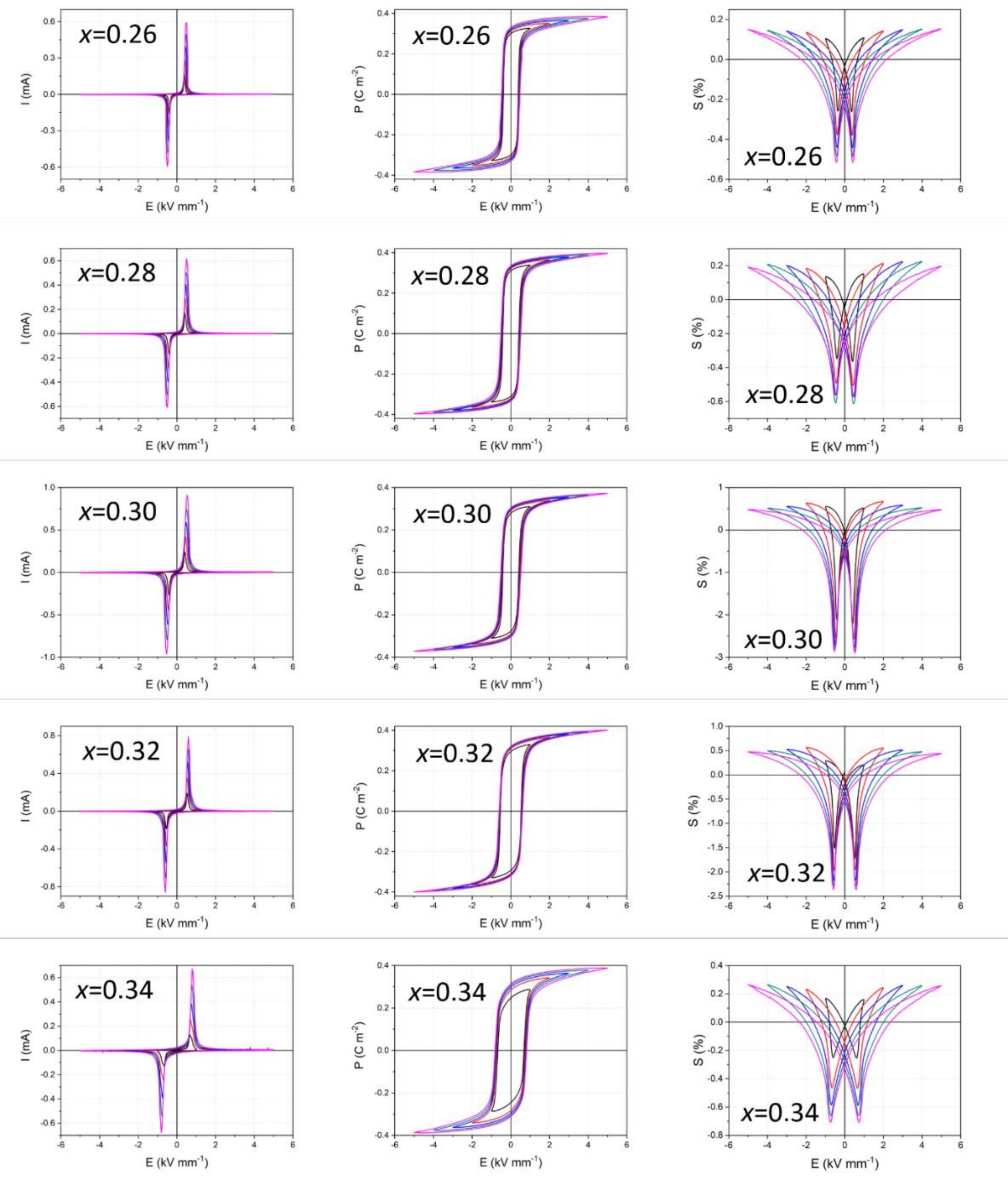

Supplementary Fig. 5 Electric field dependence of piezoelectric coefficient d_{33}^* for compositions in the system $Er_{0.025}Pb_{0.9625}(Mg_{0.33}Nb_{0.67})_{1-x}Ti_xO_3$ at room temperature: (a) bipolar and **(b)** unipolar

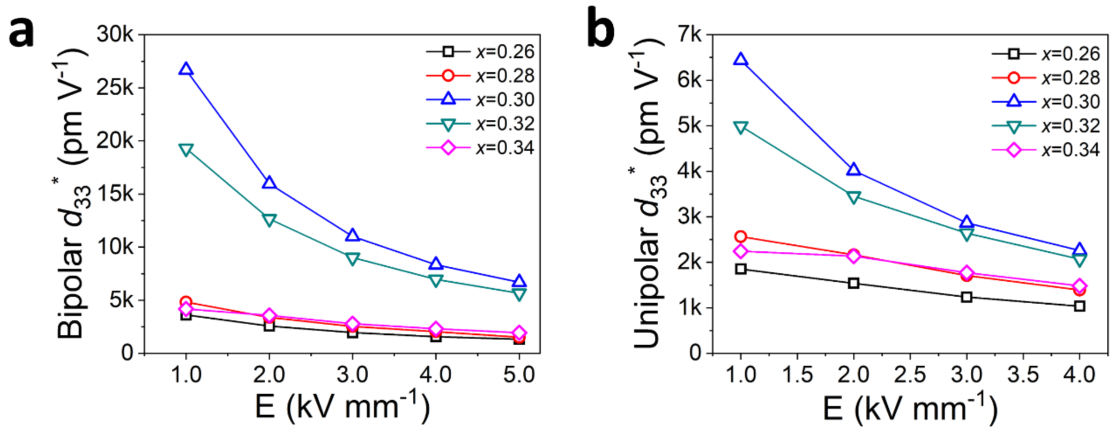

Supplementary Fig. 6 Electric field induced unipolar strain at room temperature for compositions in the system $Er_{0.025}Pb_{0.9625}(Mg_{0.33}Nb_{0.67})_{1-x}Ti_xO_3$: (**a**) $x = 0.26$, (**b**) $x = 0.28$, (**c**) $x = 0.30$, (**d**) $x = 0.32$ and (**e**) $x = 0.34$.

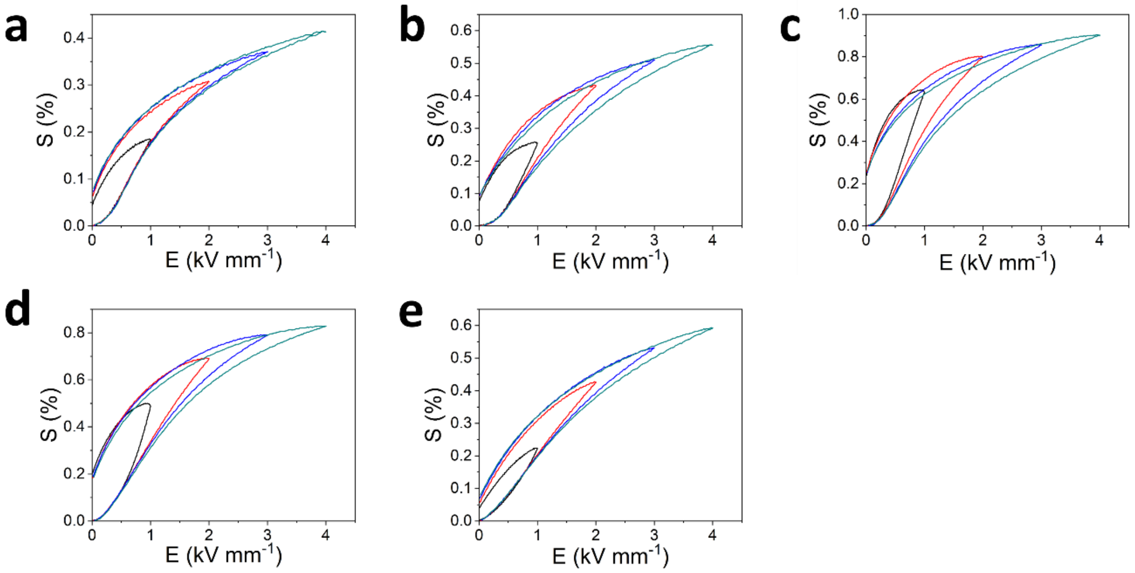

Supplementary Fig. 7 Electric field induced cyclic strain in the *x* = 0.30 composition: **(a, b)** unipolar and **(c, d)** bipolar **(a, c)** before and **(b, d)** after 1000 cycles.

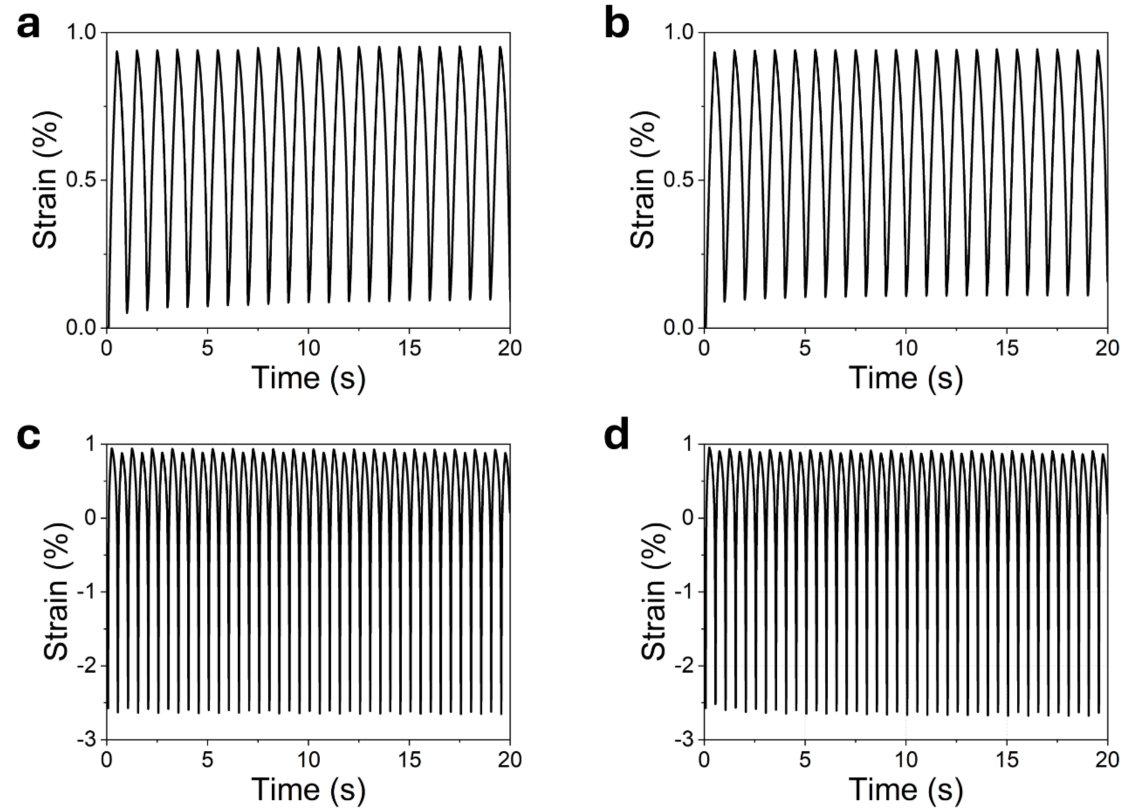

Supplementary Fig. 8 Frequency dependence of dielectric permittivity and loss for samples of the x = 0.30 composition of different thickness (mm) in the system $Er_{0.025}Pb_{0.9625}(Mg_{0.33}Nb_{0.67})_{1-x}Ti_xO_3$.

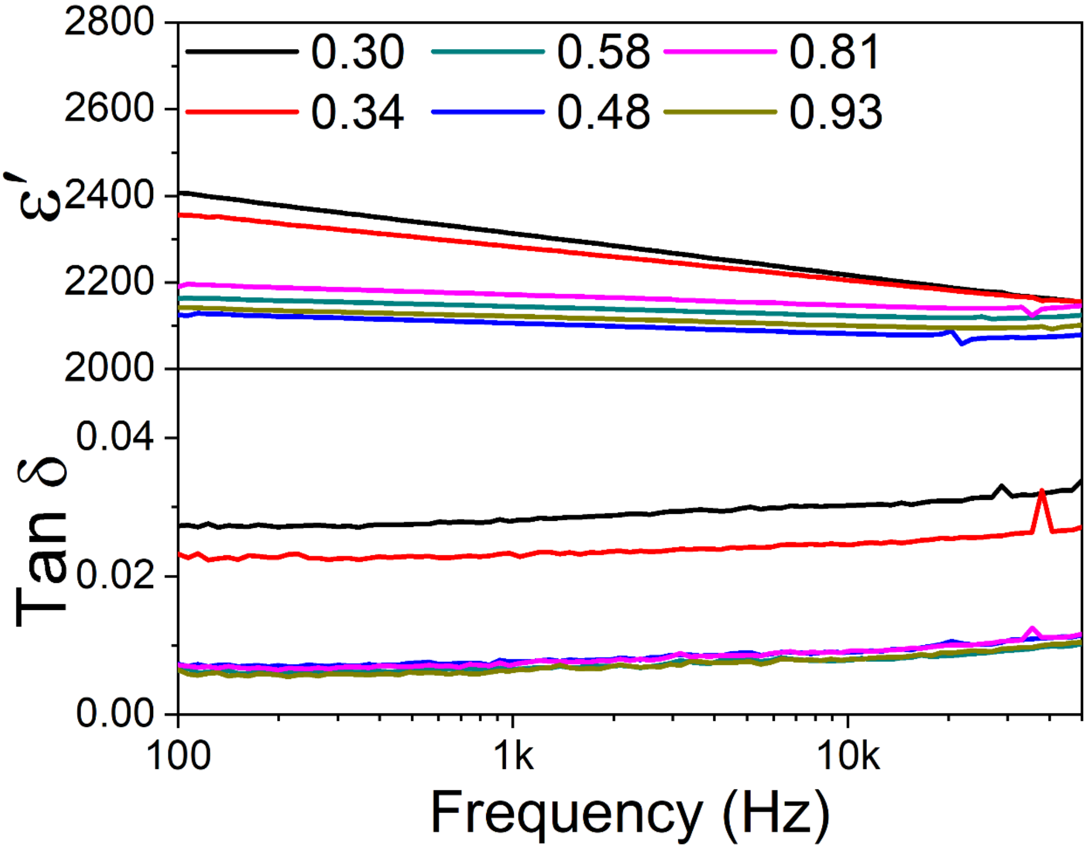

Supplementary Fig. 9 Displacement (Δ*l*) under (a) bipolar and (b) unipolar electric field at room temperature for samples of the $x = 0.30$ composition of different thickness (mm) in the system $Er_{0.025}Pb_{0.9625}(Mg_{0.33}Nb_{0.67})_{1-x}Ti_xO_3$.

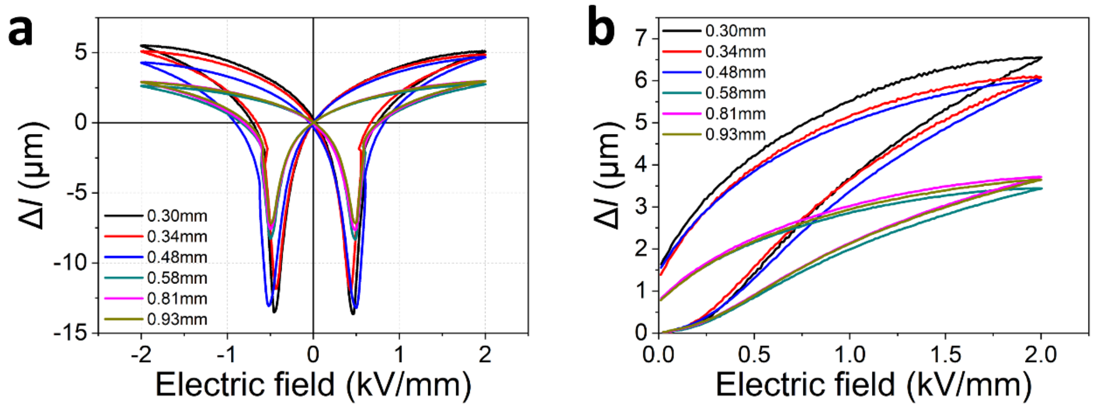

Supplementary Fig. 10 Thickness dependence of Vickers hardness with error bars (standard error) for the x = 0.30 composition in the system $Er_{0.025}Pb_{0.9625}(Mg_{0.33}Nb_{0.67})_{1-x}Ti_xO_3$. Scale bar is 10 μm.

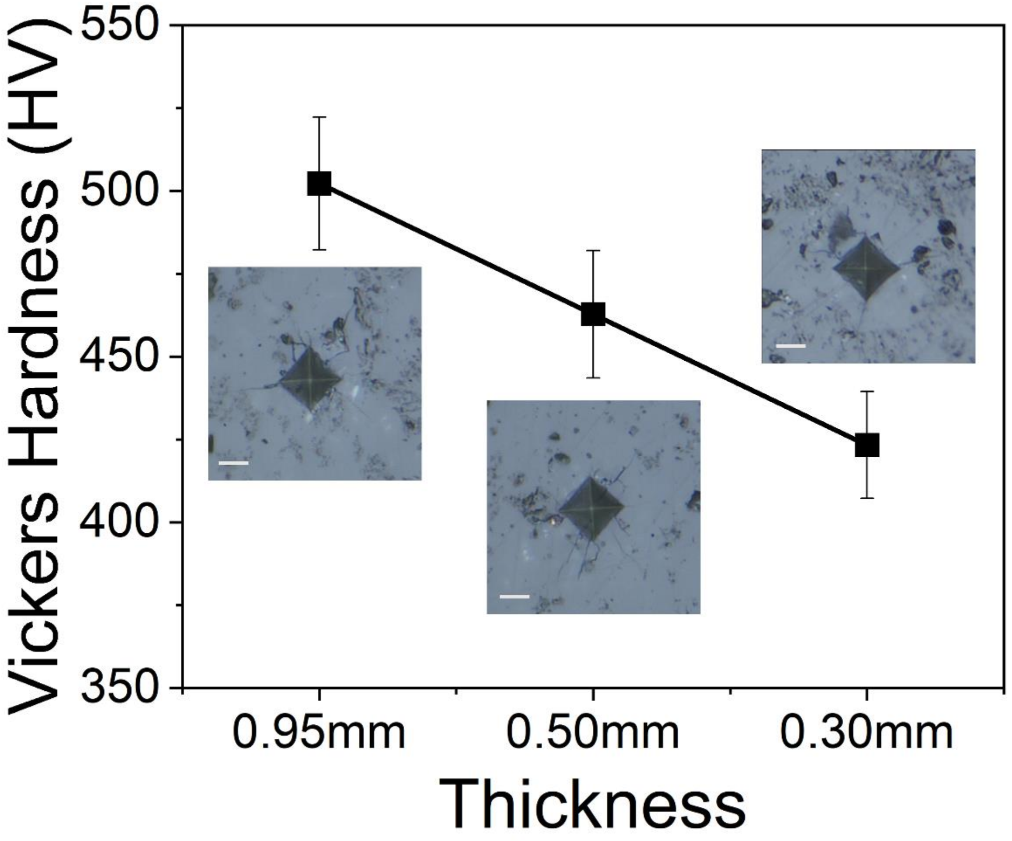

Supplementary Fig. 11 *I-E* and *P-E* loops for the x = 0.30 composition in the system $Er_{0.025}Pb_{0.9625}(Mg_{0.33}Nb_{0.67})_{1-x}Ti_xO_3$ measured at selected temperatures. At temperatures above 150 °C, which is above T_c, and the system is nominally in the paraelectric state, the presence of current peaks indicates the existence of PNRs within the material. As the temperature was further increased, the concentration of PNRs decreased, resulting in decreases in polarisation and the magnitude of the current peaks.

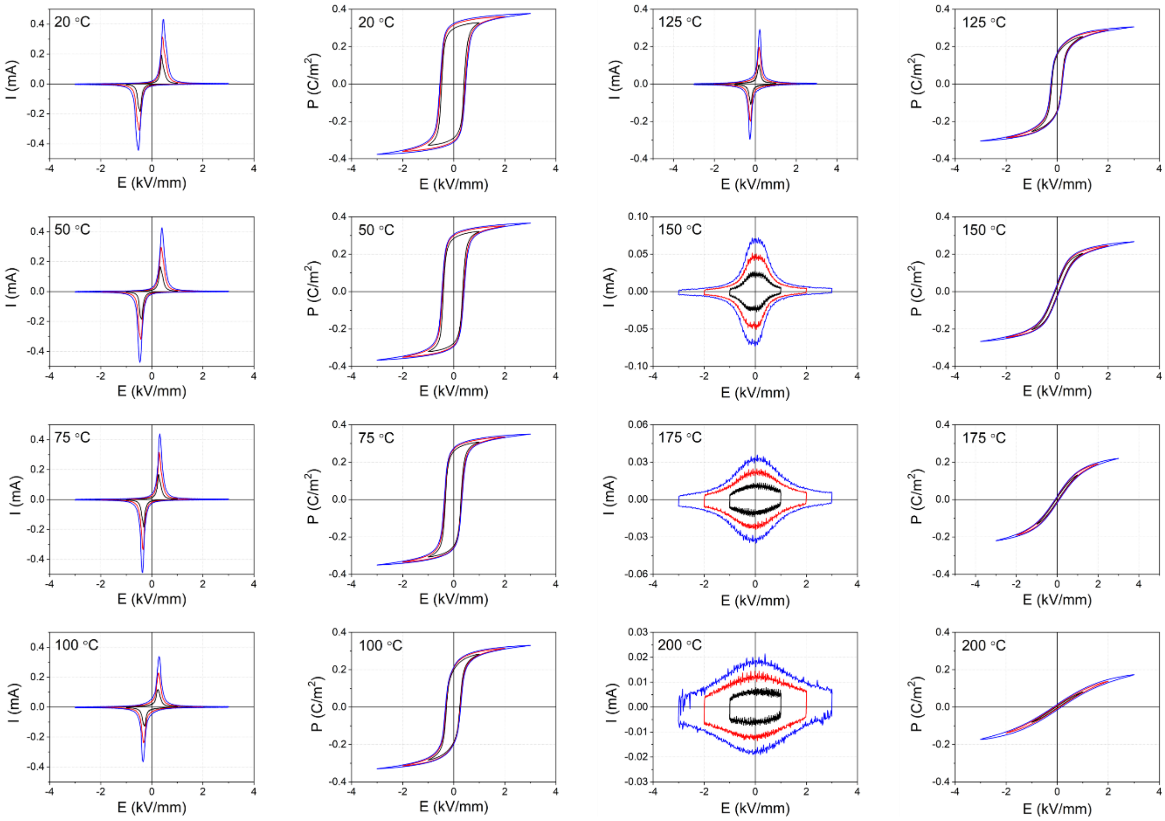

Supplementary Fig. 12 Temperature dependence of saturation polarisation (P_s) and remanent polarisation (P_r) for the x = 0.30 composition in the $Er_{0.025}Pb_{0.9625}(Mg_{0.33}Nb_{0.67})_{1-x}Ti_xO_3$ system.

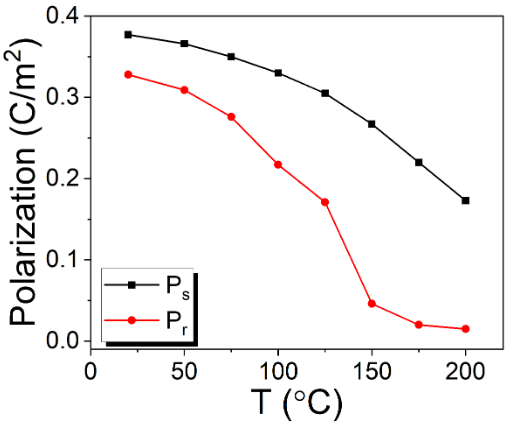

Supplementary Fig. 13 XRD patterns of unpoled and poled ceramics for the x = 0.32 (black solid line) and x = 0.34 (red solid line) compositions in the $Er_{0.025}Pb_{0.9625}(Mg_{0.33}Nb_{0.67})_{1-x}Ti_xO_3$ system including O and T phase fractions.

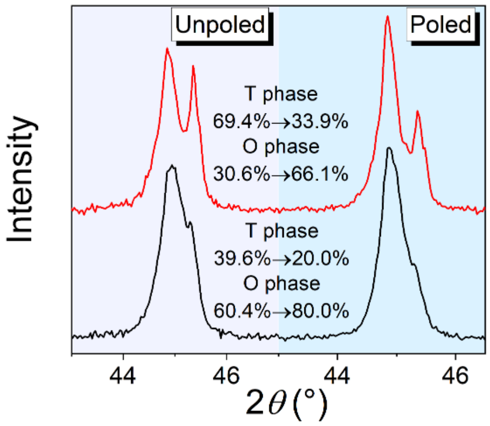

Supplementary Fig. 14 Temperature dependence of dielectric properties for unpoled and poled samples of the x = 0.30 composition in the $Er_{0.025}Pb_{0.9625}(Mg_{0.33}Nb_{0.67})_{1-x}Ti_xO_3$ system.

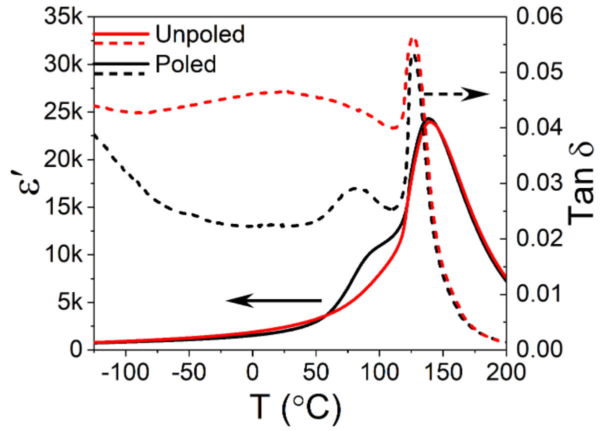

Supplementary Fig. 15 (a) Temperature dependence of dielectric permittivity and loss for the x = 0.30 composition in the system $Er_{0.025}Pb_{0.9625}(Mg_{0.33}Nb_{0.67})_{1-x}Ti_xO_3$ and (b) Curie-Weiss fit to dielectric permittivity for the x = 0.30 composition. To determine the Burns temperature, T_B, the point at which PNRs are formed, high temperature dielectric permittivity and loss data for the $x = 0.30$ sample were collected. The dielectric permittivity measured at 100 kHz was fitted using the Curie Weiss law [5]: $\frac{1}{\varepsilon'} = \frac{T - T_{CW}}{C}$, where T_{CW} is the Curie-Weiss temperature and C is the Curie constant. The fit shows that the dielectric permittivity starts to deviate from the Curie Weiss law at around 270 °C corresponding to T_B. At temperatures above T_B the pure paraelectric phase is obtained.

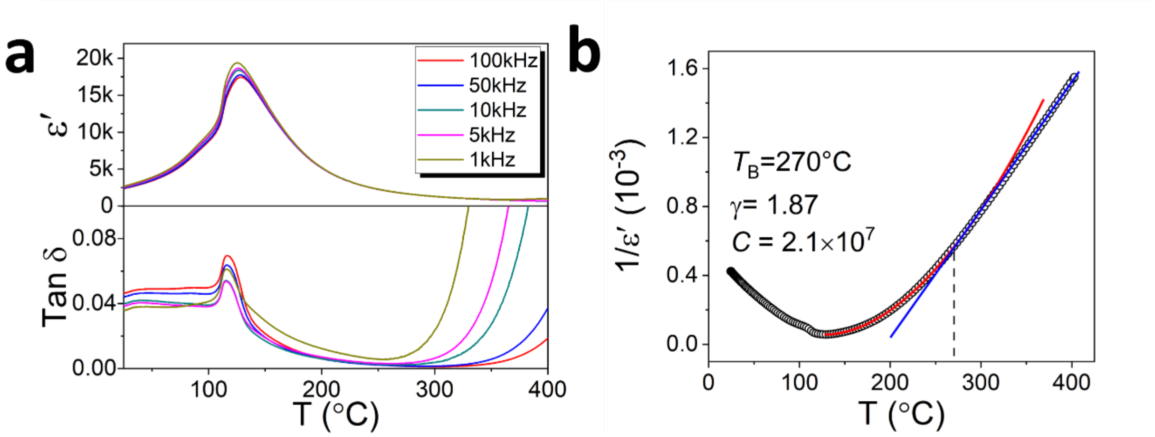

Supplementary Fig. 16 Electric field dependence of structure of the of x = 0.30 composition: (**a**) XRD pattens under selected DC electric fields with detail of the $(111)_{pc}$ peak in (**b**); (**c**) DC field variation of O and T phase lattice parameters.

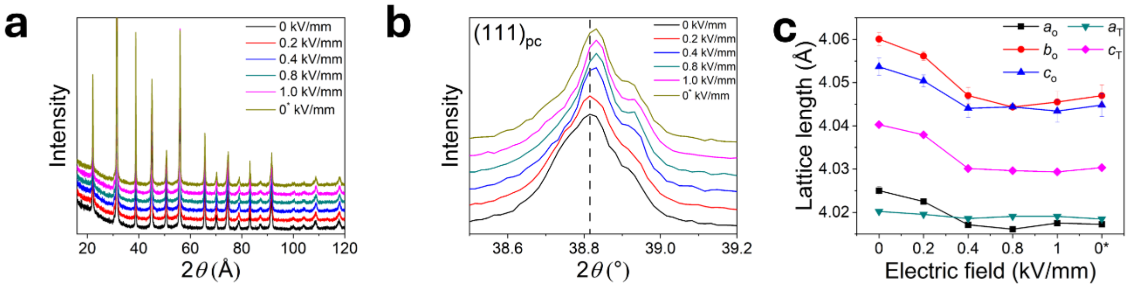

0*: 0V after applied electric field.

Supplementary Fig. 17 PFM (a) amplitude and (b) phase changes under applied external electric field for the x = 0.30 composition in the system $Er_{0.025}Pb_{0.9625}(Mg_{0.33}Nb_{0.67})_{1-x}Ti_xO_3$.

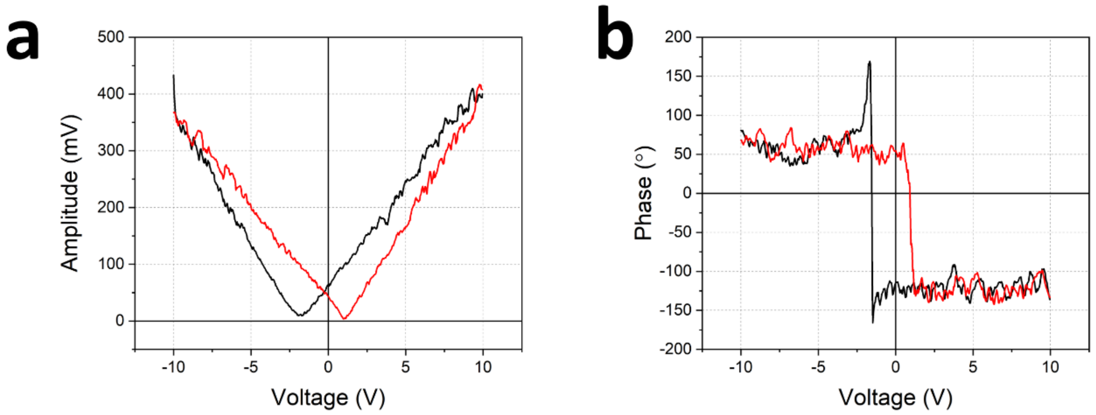

Supplementary Fig. 18 Fitted Raman spectra for unpoled and poled ceramic samples of compositions in the system $Er_{0.025}Pb_{0.9625}(Mg_{0.33}Nb_{0.67})_{1-x}Ti_xO_3$, with individual mode fits. The Raman spectra were deconvoluted into nine Raman modes and fitted using a Lorentzian peak function. The Raman modes can be categorized into three distinct regions: *A*) low wavenumber range (< 200 cm^{-1}, A_1) associated with the movement of A-site cations, *B*) mid-range wavenumbers (200 - 400 cm^{-1}, B_1, B_2, and B_3) corresponding to B-O vibrations and *C*) high wavenumber range (>400 cm^{-1}, C_1, C_2, C_3, C_4 and C_5) associated with stretching and breathing modes in the BO_6 octahedra. The observation of multiple Raman peaks in the *B* (wavenumber of 200 - 400 cm^{-1}) and *C* (wavenumber above 400 cm^{-1}) regions indicates the presence of a mixture of phases [6].

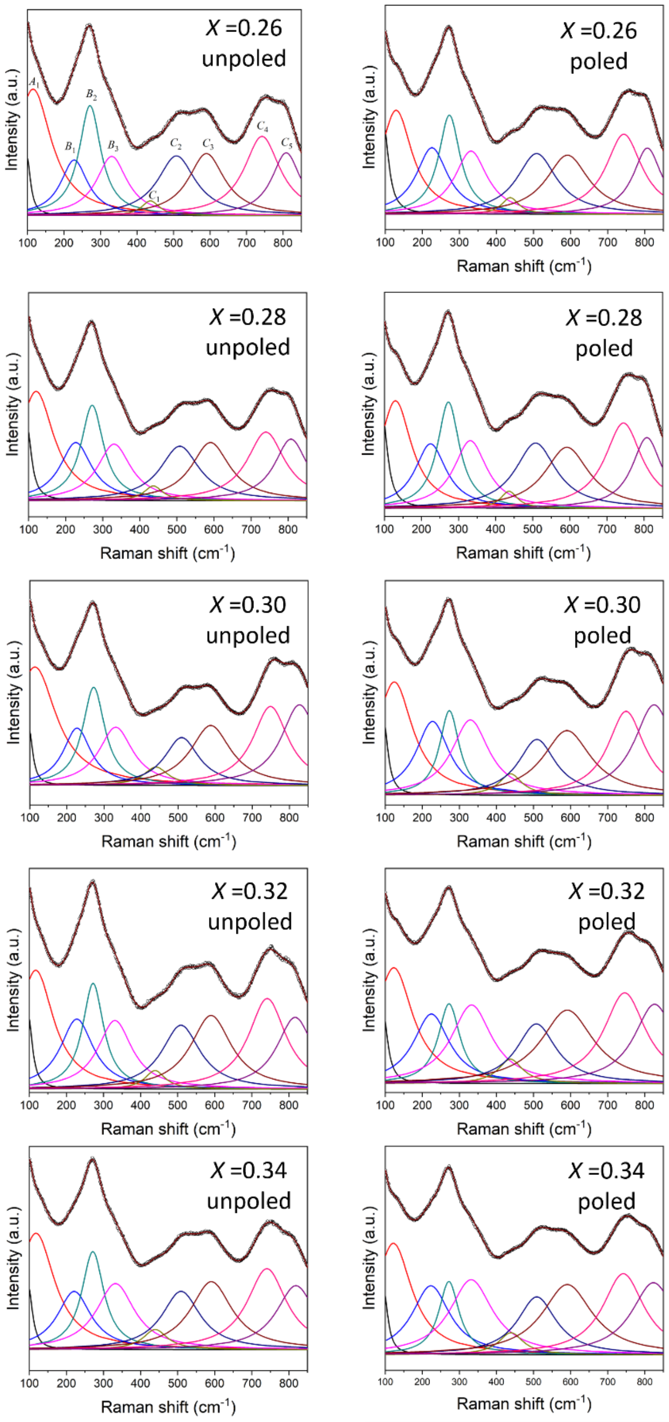

Supplementary Fig. 19 Compositional variation with error bars ((standard error) of characteristic Raman shifts in the $Er_{0.025}Pb_{0.9625}(Mg_{0.33}Nb_{0.67})_{1-x}Ti_xO_3$ system.

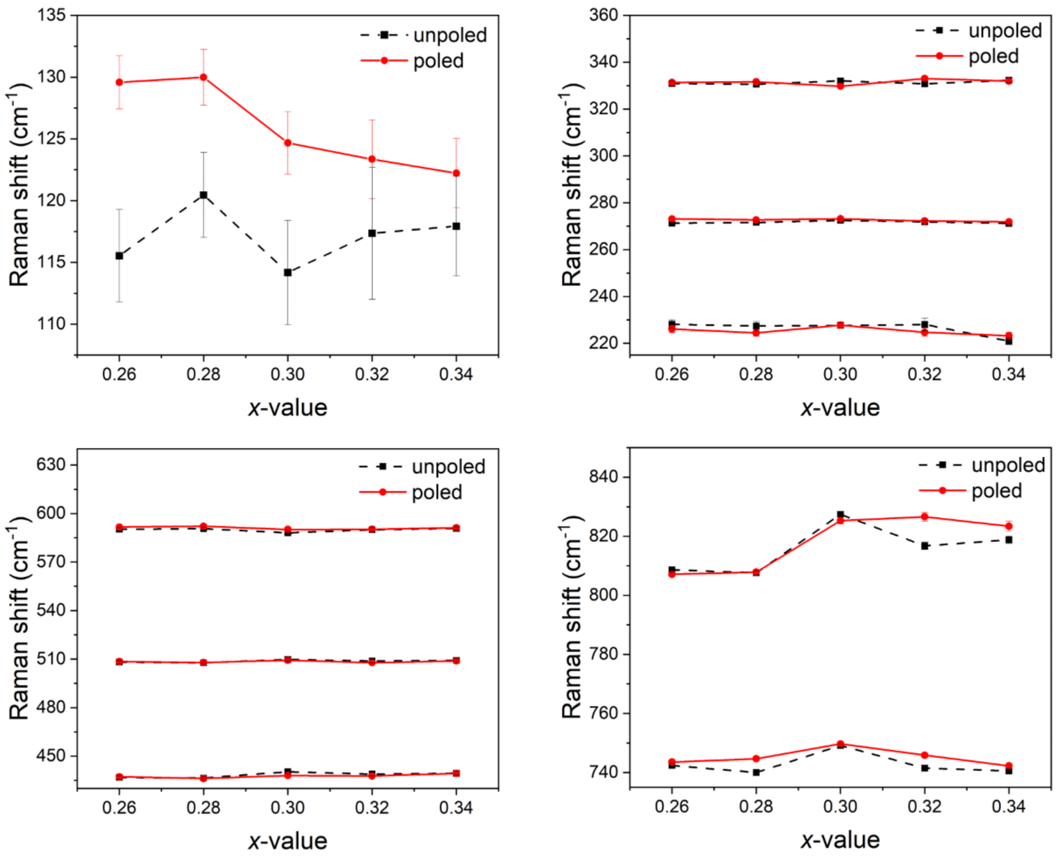

Supplementary Fig. 20 (a) Raman shift difference and (b) intensity ratio of C_3 and C_2 modes for studied compositions in the $Er_{0.025}Pb_{0.9625}(Mg_{0.33}Nb_{0.67})_{1-x}Ti_xO_3$ system.

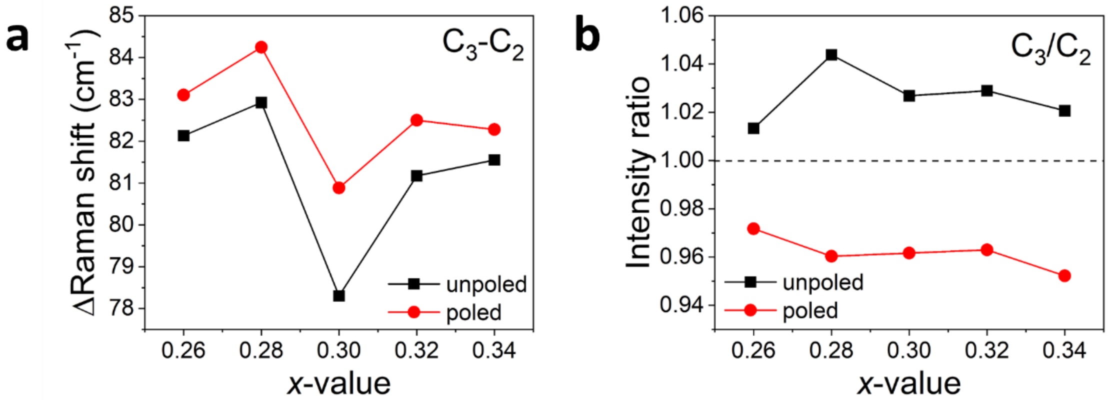

Supplementary Fig. 21 (a) High resolution TEM image and (b-g) Energy-Dispersive X-ray Spectroscopy (EDS) images of the x = 0.30 composition in the $Er_{0.025}Pb_{0.9625}(Mg_{0.33}Nb_{0.67})_{1-x}Ti_xO_3$ system, showing the distribution of (b) Er, (c) Pb, (d) Mg, (e) Nb, (f) Ti, and (g) O.

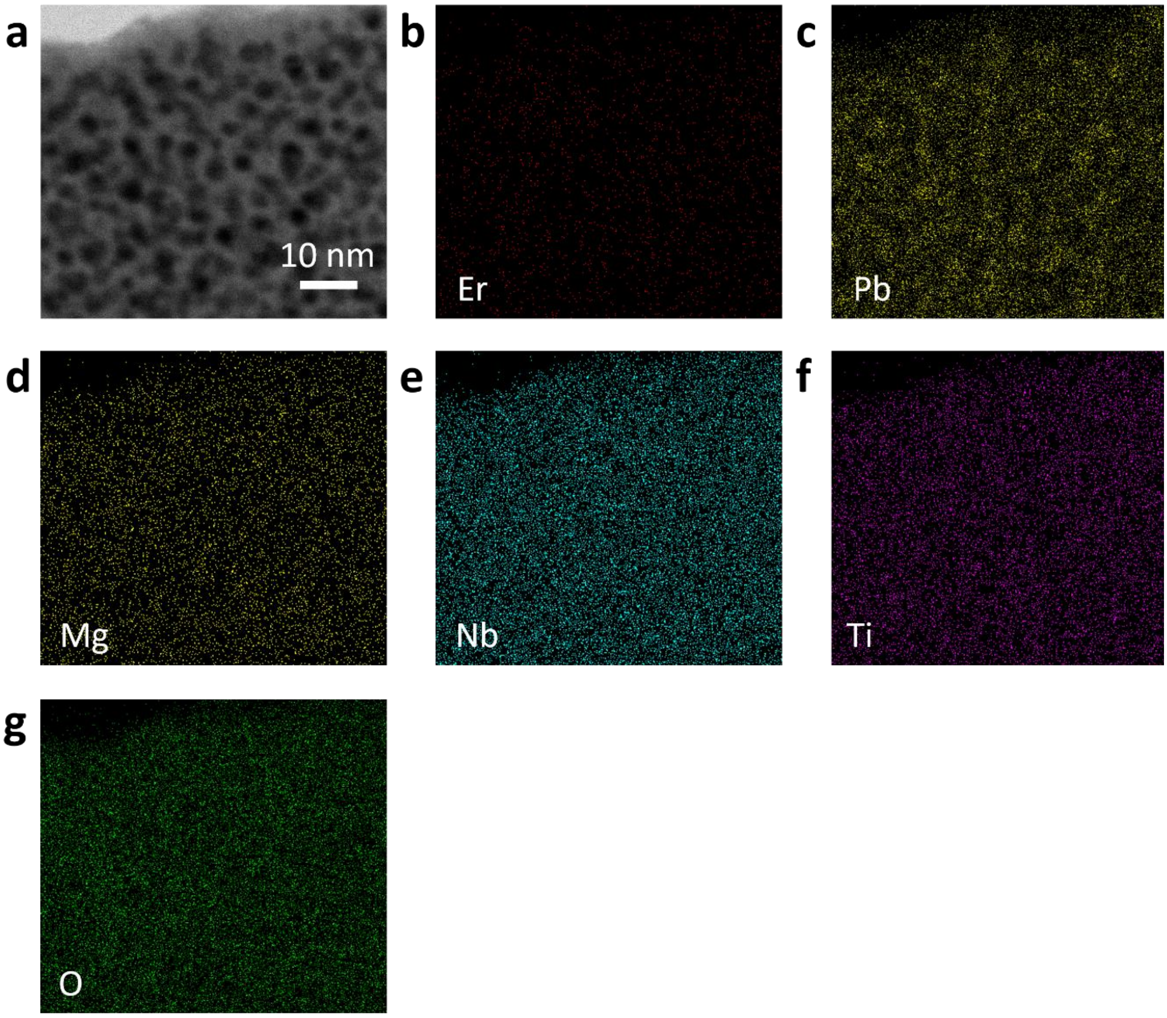

Supplementary Fig. 22 SAED images of the x = 0.30 composition in the system $Er_{0.025}Pb_{0.9625}(Mg_{0.33}Nb_{0.67})_{1-x}Ti_xO_3$ along (a) [100], (b) [010], (c) [10-3] and (d) [221] zone axes.

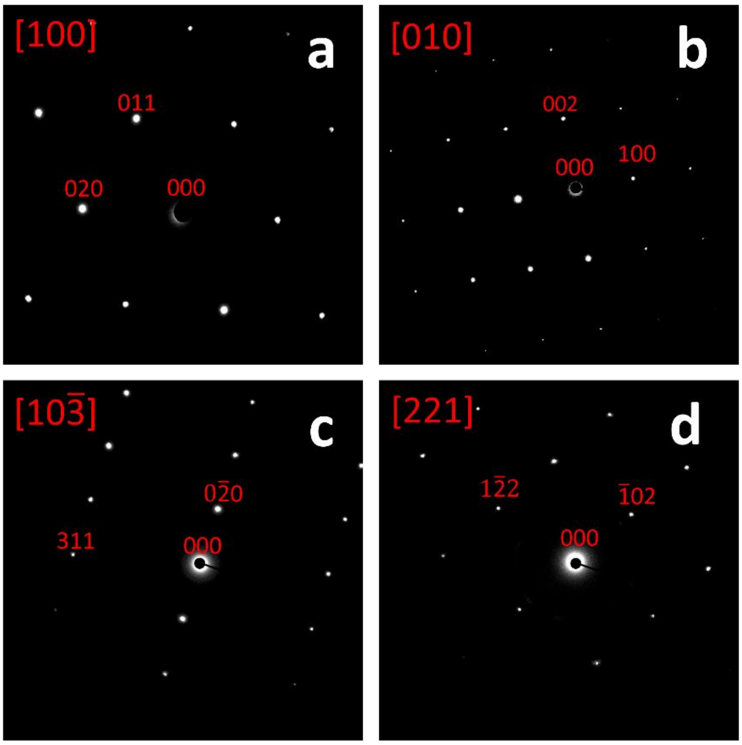

Supplementary Fig. 23 (a) Conventional and (b) high resolution TEM images of the *x* = 0.30 composition in the $Er_{0.025}Pb_{0.9625}(Mg_{0.33}Nb_{0.67})_{1-x}Ti_xO_3$ system.

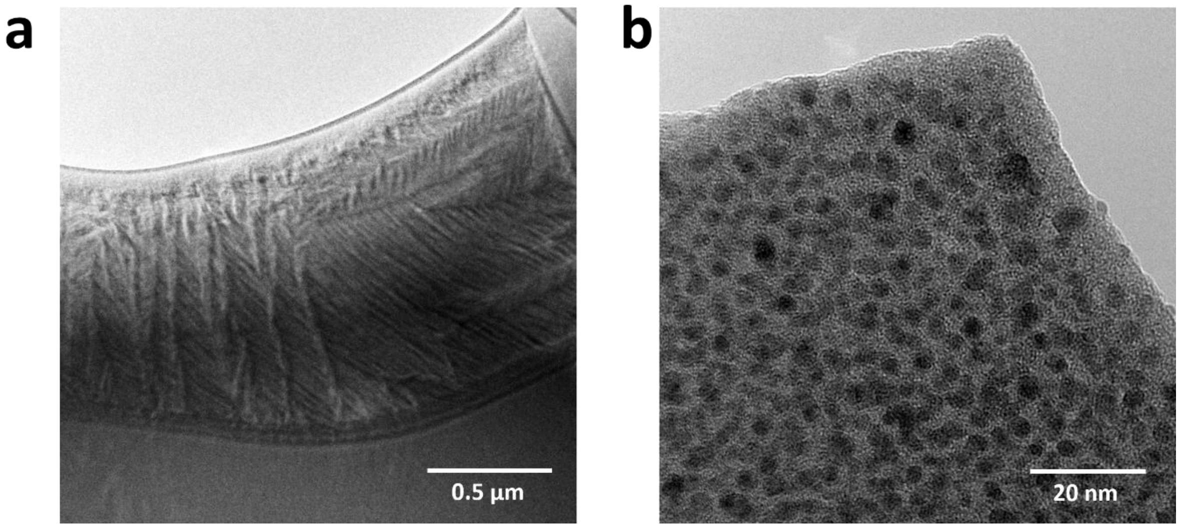

Supplementary Fig. 24 Schematic diagrams of setups used for (a) XRD measurements under DC electric field and (b) S-E loop measurements. Recent studies have reported ultra-high strain; however, in many cases, this strain has been attributed to bending effects[7–10]. High strain caused by bending is typically associated with asymmetric S-E loops. In contrast, our S-E loops for samples of varying thickness are symmetric, strongly indicating that the high strain observed in our samples is not due to bending deformation. The setup used for measuring the S-E loops is shown in (b). In this configuration, the ceramic sample was placed between a spherical top electrode and a flat bottom electrode. According to He *et al.*, [10] such a setup would produce asymmetric S-E loops if bending deformation were a significant factor. Therefore, the symmetry of the S-E loops observed in our study confirms that the high strain is intrinsic to the material rather than a result of bending effects.

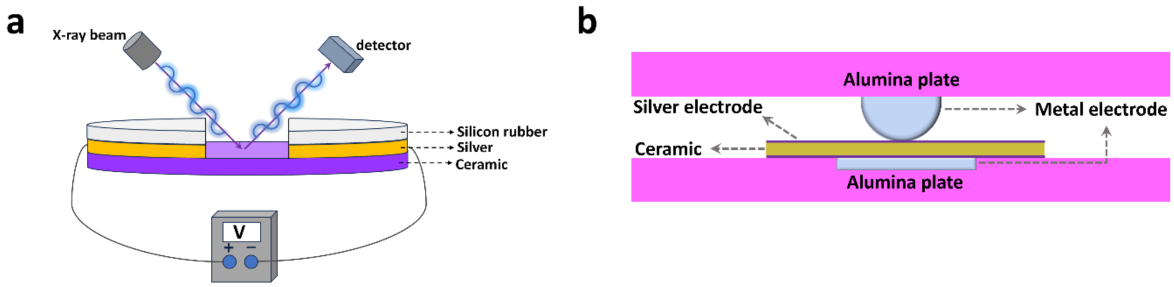

Supplementary Table 1. Crystal and refinement parameters derived from Rietveld analysis of XRD data for compositions in the $Er_{0.025}Pb_{0.9625}(Mg_{0.33}Nb_{0.67})_{1-x}Ti_xO_3$ system at room temperature. Estimated standard deviations are given in parentheses.

x	**0.26**	**0.28**	**0.30**	**0.32**		**0.34**	
**Space group**	*Amm*2	*Amm*2	*Amm*2	*P*4*mm*	*Amm*2	*P*4*mm*	*Amm*2
**Weight fraction**	1	1	1	0.512(5)	0.487(5)	0.747(2)	0.252(6)
**Unit cell dimensions (Å)**	a = 4.0265(1)<br>b = 5.6965(2)<br>c = 5.7086(2)	a = 4.0217(1)<br>b = 5.6968(2)<br>c = 5.7074(3)	a = 4.0190(1)<br>b = 5.6958(2)<br>c = 5.7073(2)	a = 4.0127(1)<br>c = 4.0491(1)	a = 4.0135(2)<br>b = 5.6973(4)<br>c = 5.7099(4)	a = 4.00772(6)<br>c = 4.05240(8)	a = 4.0114(6)<br>b = 5.696(1)<br>c = 5.713(1)
**Volume (Å^3)**	130.940(7)	130.764(8)	130.650(8)	65.198(4)	130.56(1)	65.089(3)	130.55(2)
**Z**	2	2	2	1	2	1	2
**D_{calc} (g cm^{-3})**	8.012	8.012	8.007	8.012	8.001	8.014	7.991
**R-factors**	R_{wp} = 0.0436<br>R_p = 0.0321<br>R_{ex} =0.0233<br>R_F^2 = 0.0599<br>χ^2 = 3.543	R_{wp} = 0.0432<br>R_p = 0.0317<br>R_{ex} =0.0233<br>R_F^2 = 0.0699<br>χ^2 = 3.762	R_{wp} = 0.0413<br>R_p = 0.0306<br>R_{ex} =0.0239<br>R_F^2 = 0.0656<br>χ^2 = 3.013	R_{wp} = 0.0467<br>R_p = 0.0339<br>R_{ex} =0.0234<br>R_F^2 = 0.0832<br>χ^2 = 4.009		R_{wp} = 0.0473<br>R_p = 0.0337<br>R_{ex} =0.0237<br>R_F^2 = 0.1094<br>χ^2 = 4.019	
**No. of variables**	25	25	25	27		27	
**No. of profile points**	3649	3649	3649	3649		3649	

Supplementary Table 2. Atomic coordinates and isotropic thermal parameters derived from Rietveld analysis of XRD data for compositions in the $Er_{0.025}Pb_{0.9625}(Mg_{0.33}Nb_{0.67})_{1-x}Ti_xO_3$ system at room temperature. Estimated standard deviations are given in parentheses.

				**0.26**		
**(*Amm*2) Atom**	**Site**	***x***	***y***	***z***	**Occ.**	**U_{iso} (Å^2)**
Pb/Er	2a	0.0	0.0	0.0	0.9625/0.025	0.042(1)
Mg/Nb/Ti	2b	0.5	0.0	0.524(2)	0.493/0.247/0.260	0.012(1)
O1	2a	0.0	0.0	0.0489	1.00	0.057(2)
O2	2e	0.5	0.2561	0.2333	1.00	0.057(2)
				**0.28**		
**(*Amm*2) Atom**	**Site**	***x***	***y***	***z***	**Occ.**	**U_{iso} (Å^2)**
Pb/Er	2a	0.0	0.0	0.0	0.9625/0.025	0.048(1)
Mg/Nb/Ti	2b	0.5	0.0	0.530(2)	0.493/0.247/0.280	0.012(1)
O1	2a	0.0	0.0	0.0489	1.00	0.050(2)
O2	2e	0.5	0.2561	0.2333	1.00	0.050(2)
				**0.30**		
**(*Amm*2) Atom**	**Site**	***x***	***y***	***z***	**Occ.**	**U_{iso} (Å^2)**
Pb/Er	2a	0.0	0.0	0.0	0.9625/0.025	0.039(1)
Mg/Nb/Ti	2b	0.5	0.0	0.530(2)	0.480/0.240/0.280	0.007(1)
O1	2a	0.0	0.0	0.0489	1.00	0.044(2)
O2	2e	0.5	0.2561	0.2333	1.00	0.044(2)
				**0.32**		
**(*Amm*2) Atom**	**Site**	***x***	***y***	***z***	**Occ.**	**U_{iso} (Å^2)**
Pb/Er	2a	0.0	0.0	0.0	0.9625/0.025	0.0394(4)
Mg/Nb/Ti	2b	0.5	0.0	0.536125	0.453/0.227/0.32	0.0114(7)
O1	2a	0.0	0.0	0.0489	1.00	0.056(2)
O2	2e	0.5	0.2561	0.2333	1.00	0.056(2)
**(*P*4*mm*) Atom**	**Site**	***x***	***y***	***z***	**Occ.**	**U_{iso} (Å^2)**
Pb/Er	1a	0.0	0.0	0.0	0.9625/0.025	0.0394(4)
Mg/Nb/Ti	1b	0.5	0.5	0.527464	0.453/0.227/0.320	0.0114(7)
O1	1b	0.5	0.5	0.038	1.00	0.056(2)
O2	1c	0.5	0.0	0.5495	1.00	0.056(2)
				**0.34**		
**(*Amm*2) Atom**	**Site**	***x***	***y***	***z***	**Occ.**	**U_{iso} (Å^2)**
Pb/Er	2a	0.0	0.0	0.0	0.9625/0.025	0.0361(4)
Mg/Nb/Ti	2b	0.5	0.0	0.536125	0.440/0.220/0.340	0.0093(7)
O1	2a	0.0	0.0	0.0489	1.00	0.024(2)
O2	2e	0.5	0.2561	0.2333	1.00	0.024(2)
**(*P*4*mm*) Atom**	**Site**	***x***	***y***	***z***	**Occ.**	**U_{iso} (Å^2)**
Pb/Er	1a	0.0	0.0	0.0	0.9625/0.025	0.0361(4)
Mg/Nb/Ti	1b	0.5	0.5	0.527464	0.440/0.220/0.340	0.0093(7)
O1	1b	0.5	0.5	0.038	1.00	0.024(2)
O2	1c	0.5	0.0	0.5495	1.00	0.024(2)

Supplementary Table 3. Crystal and refinement parameters for $Er_{0.025}Pb_{0.9625}Mg_{0.4667}Nb_{0.2333}Ti_{0.3}O_3$ at (a) -173 °C, (b) room temperature, (c) 120 °C and (d) 200 °C based on fits to high-resolution neutron diffraction data. Estimated standard deviations are given in parentheses.

(a)		
**$Er_{0.025}Pb_{0.9625}Mg_{0.4667}Nb_{0.2333}Ti_{0.3}O_3$ @ -173 °C**	*P*4*mm* model	*Amm*2 model
**Weight Fraction**	64.1(4)%	35.9(5)%
**Unit cell dimensions**	*a* = 4.01669(3) Å<br>*c* = 4.024747(5) Å	*a* = 4.0296(1) Å<br>*b* = 5.6961(2) Å<br>*c* = 5.7116(1) Å
**Volume (Å^3)**	64.934(1)	131.101(6)
**Z**	1	2
**D_{calc} (g cm^{-3})**	8.056	7.980
**R-factors**	R_{wp} = 0.0451<br>R_p = 0.0410<br>R_{ex} = 0.0210<br>R_F^2 = 0.0558	
**No. of variables**	80	
**No. of profile points**	4407	

(b)		
**$Er_{0.025}Pb_{0.9625}Mg_{0.4667}Nb_{0.2333}Ti_{0.3}O_3$ @ 22 °C**	*P*4*mm* model	*Amm*2 model
**Weight Fraction**	71.8(4)%	28.2(5)%
**Unit cell dimensions**	*a* = 4.02061(2) Å<br>*c* = 4.02680(3) Å	*a* = 4.0315(1) Å<br>*b* = 5.6983(2) Å<br>*c* = 5.7029(1) Å
**Volume (Å^3)**	65.095(1)	131.013(5)
**Z**	1	2
**D_{calc} (g cm^{-3})**	8.036	7.985
**R-factors**	R_{wp} = 0.0371<br>R_p = 0.0320<br>R_{ex} = 0.0210<br>R_F^2 = 0.0505	
**No. of variables**	80	
**No. of profile points**	4407	

(c)		
**$Er_{0.025}Pb_{0.9625}Mg_{0.4667}Nb_{0.2333}Ti_{0.3}O_3$ @ 120 °C**	*P*4*mm* model	*Pm*-3*m* model
**Weight Fraction**	22.7(4)%	77.3(2)%
**Unit cell dimensions**	*a* = 4.02030(3) Å<br>*c* = 4.03518(7) Å	*a* = 4.025639(7) Å
**Volume (Å^3)**	65.220(1)	65.239(0)
**Z**	1	1
**D_{calc} (g cm^{-3})**	8.020	8.018
**R-factors**	R_{wp} = 0.0369<br>R_p = 0.0354<br>R_{ex} = 0.0250<br>R_F^2 = 0.0355	
**No. of variables**	65	
**No. of profile points**	4407	

(d)	
**$Er_{0.025}Pb_{0.9625}Mg_{0.4667}Nb_{0.2333}Ti_{0.3}O_3$ @ 200 °C**	*Pm*-3*m* model
**Unit cell dimensions**	*a* = 4.025639(7) Å
**Volume (Å^3)**	65.239(0)
**Z**	1
**D_{calc} (g cm^{-3})**	8.013
**R-factors**	R_{wp} = 0.0420<br>R_p = 0.0385<br>R_{ex} = 0.0251<br>R_F^2 = 0.0282
**No. of variables**	46
**No. of profile points**	4407

Supplementary Table 4. Refined structural parameters for $Er_{0.025}Pb_{0.9625}Mg_{0.4667}Nb_{0.2333}Ti_{0.3}O_3$ based on fits to high-resolution neutron diffraction data using *Amm*2, *P*4*mm* and *Pm*-3*m* models. Estimated standard deviations are given in parentheses.

$Er_{0.025}Pb_{0.9625}Mg_{0.4667}Nb_{0.2333}Ti_{0.3}O_3$ @ -173 °C

						P4mm					
**Atom**	**Site**	***x***	***y***	***z***	**Occ.**	**U_{11} (Å^2)**	**U_{22} (Å^2)**	**U_{33} (Å^2)**	**U_{12} (Å^2)**	**U_{13} (Å^2)**	**U_{23} (Å^2)**
Pb/Er	1a	0.0	0.0	0.0	0.9625/0.025	0.0383(9)	0.0383(9)	0.0343(3)	0.0	0.0	0.0
Mg/Nb/Ti	1b	0.5	0.5	0.532(1)	0.233/0.467/0.3	0.0030(9)	0.0030(9)	0.009(2)	0.0	0.0	0.0
O1	1b	0.5	0.5	0.051(1)	1.0	0.008(1)	0.008(6)	0.033(2)	0.0	0.0	0.0
O2	2c	0.5	0	0.5599(8)	1.0	0.028(1)	0.0043(9)	0.013(1)	0.0	0.0	0.0
						*Amm*2					
**Atom**	**Site**	***x***	***y***	***z***	**Occ.**	**U_{11} (Å^2)**	**U_{22} (Å^2)**	**U_{33} (Å^2)**	**U_{12} (Å^2)**	**U_{13} (Å^2)**	**U_{23} (Å^2)**
Pb/Er	2a	0.0	0.0	0.0	0.9625/0.025	0.027(2)	0.113(5)	0.0002(11)	0.0	0.0	0.0
Mg/Nb/Ti	2b	0.5	0.5	0.4637(6)	0.233/0.467/0.3	0.012(3)	0.004(3)	0.001(3)	0.0	0.0	0.0
O1	2a	0.5	0.5	0.4375(9)	1.00	0.063(4)	-0.020(1)	0.001(1)	0.0	0.0	0.0
O2	4e	0.0	0.2115(7)	0.1771(6)	1.00	0.010(2)	0.035(3)	0.002(1)	0.0	0.0	0.017(1)

$Er_{0.025}Pb_{0.9625}Mg_{0.4667}Nb_{0.2333}Ti_{0.3}O_3$ @ 22 °C

						P4mm					
**Atom**	**Site**	***x***	***y***	***z***	**Occ.**	**U_{11} (Å^2)**	**U_{22} (Å^2)**	**U_{33} (Å^2)**	**U_{12} (Å^2)**	**U_{13} (Å^2)**	**U_{23} (Å^2)**
Pb/Er	1a	0.0	0.0	0.0	0.9625/0.025	0.0380(7)	0.0380(7)	0.050(3)	0.0	0.0	0.0
Mg/Nb/Ti	1b	0.5	0.5	0.529(2)	0.233/0.467/0.3	0.0064(7)	0.0064(7)	0.006(1)	0.0	0.0	0.0
O1	1b	0.5	0.5	0.042(1)	1.0	0.025(1)	0.025(6)	0.030(1)	0.0	0.0	0.0
O2	2c	0.5	0	0.5483(9)	1.0	0.020(1)	0.0074(7)	0.022(1)	0.0	0.0	0.0
						*Amm*2					
**Atom**	**Site**	***x***	***y***	***z***	**Occ.**	**U_{11} (Å^2)**	**U_{22} (Å^2)**	**U_{33} (Å^2)**	**U_{12} (Å^2)**	**U_{13} (Å^2)**	**U_{23} (Å^2)**
Pb/Er	2a	0.0	0.0	0.0	0.9625/0.025	0.031(4)	0.014(4)	0.008(2)	0.0	0.0	0.0
Mg/Nb/Ti	2b	0.5	0.5	0.457(1)	0.233/0.467/0.3	0.018(5)	0.018(5)	-0.023(4)	0.0	0.0	0.0
O1	2a	0.5	0.5	0.433(2)	1.00	0.030(5)	0.007(5)	-0.003(3)	0.0	0.0	0.0
O2	4e	0.0	0.231(1)	0.178(1)	1.00	0.012(2)	0.039(5)	0.018(4)	0.0	0.0	0.037(3)

$Er_{0.025}Pb_{0.9625}Mg_{0.4667}Nb_{0.2333}Ti_{0.3}O_3$ @ 120 °C *P4mm*

**Atom**	**Site**	***x***	***y***	***z***	**Occ.**	U_{11} **(Å^2)**	U_{22} **(Å^2)**	U_{33} **(Å^2)**	U_{12} **(Å^2)**	U_{13} **(Å^2)**	U_{23} **(Å^2)**
Pb/Er	1a	0.0	0.0	0.0	0.9625/0.025	0.034(1)	0.034(1)	0.004(2)	0.0	0.0	0.0
Mg/Nb/Ti	1b	0.5	0.5	0.546(2)	0.233/0.467/0.3	0.023(2)	0.023(2)	0.005(3)	0.0	0.0	0.0
O1	1b	0.5	0.5	0.049(2)	1.0	0.011(1)	0.011(1)	0.0305(2)	0.0	0.0	0.0
O2	2c	0.5	0	0.5483(9)	1.0	0.063(3)	0.007(1)	0.012(3)	0.0	0.0	0.0
						Pm-3m					
**Atom**	**Site**	***x***	***y***	***z***	**Occ.**	U_{11} **(Å^2)**	U_{22} **(Å^2)**	U_{33} **(Å^2)**	U_{12} **(Å^2)**	U_{13} **(Å^2)**	U_{23} **(Å^2)**
Pb/Er	1a	0.0	0.0	0.0	0.9625/0.025	0.0559(4)	0.0559(4)	0.0559(4)	0.0	0.0	0.0
Mg/Nb/Ti	1b	0.5	0.5	0.5	0.233/0.467/0.3	0.0092(3)	0.0092(3)	0.0092(3)	0.0	0.0	0.0
O	3c	0.5	0.5	0.0	1.00	0.0317(3)	0.0317(3)	0.0202(5)	0.0	0.0	0.0

$Er_{0.025}Pb_{0.9625}Mg_{0.4667}Nb_{0.2333}Ti_{0.3}O_3$ @ 200 °C *Pm-3m*

**Atom**	**Site**	***x***	***y***	***z***	**Occ.**	U_{11} **(Å^2)**	U_{22} **(Å^2)**	U_{33} **(Å^2)**	U_{12} **(Å^2)**	U_{13} **(Å^2)**	U_{23} **(Å^2)**
Pb/Er	1a	0.0	0.0	0.0	0.9625/0.025	0.0525(3)	0.0525(3)	0.0525(3)	0.0	0.0	0.0
Mg/Nb/Ti	1b	0.5	0.5	0.5	0.233/0.467/0.3	0.0119(3)	0.0119(3)	0.0119(3)	0.0	0.0	0.0
O	3c	0.5	0.5	0.0	1.00	0.0327(3)	0.0327(3)	0.0158(3)	0.0	0.0	0.0

References

1. Shimakawa, Y. *et al.* Crystal structure and ferroelectric properties of $ABi_2Ta_2O_9$ (A = Ca, Sr, and Ba). *Phys. Rev. B* **61**, 6559–6564 (2000).
2. Yan, H. *et al.* A lead-free high-curie-point ferroelectric ceramic, $CaBi_2Nb_2O_9$. *Adv. Mater.* **17**, 1261–1265 (2005).
3. Fang, B. *et al.* Temperature-dependent Raman spectra and electrical properties of $0.69Pb(Mg_{1/3}Nb_{2/3})O_3$–$0.31PbTiO_3$ single crystals. *Appl. Phys. A Mater. Sci. Process.* **122**, 1–9 (2016).
4. Huangfu, G. *et al.* Giant electric field–induced strain in lead-free piezoceramics. *Science* **378**, 1125–1130 (2022).
5. Wu, J. *et al.* Ultrahigh field-induced strain in lead-free ceramics. *Nano Energy* **76**, 105037 (2020).
6. Bokov, A. A. & Ye, Z. G. Dielectric Relaxation in Relaxor Ferroelectrics. *J. Adv. Dielectr.* **02**, 1241010 (2012).
7. Jia, Y. *et al.* Giant electro-induced strain in lead-free relaxor ferroelectrics via defect engineering. *J. Eur. Ceram. Soc.* **43**, 947–956 (2023).
8. Yan, Y. *et al.* Ultrahigh Piezoelectric Performance through Synergistic Compositional and Microstructural Engineering. *Adv. Sci.* **9**, 1–10 (2022).
9. Huangfu, G. *et al.* Giant electric field–induced strain in lead-free piezoceramics. *Science.* **378**, 1125–1130 (2022).
10. He, X. *et al.* Ultra-large electromechanical deformation in lead-free piezoceramics at reduced thickness. *Mater. Horizons* **11**, 1079–1087 (2023).
